# U.S. health professionals’ perspectives on orthorexia nervosa: clinical utility, measurement and diagnosis, and perceived influence of sociocultural factors

**DOI:** 10.1007/s40519-023-01551-6

**Published:** 2023-03-22

**Authors:** Christina M. Sanzari, Julia M. Hormes

**Affiliations:** Department of Psychology, Social Sciences 399, University at Albany, State University of New York, 1400 Washington Ave, Albany, NY 12222 USA

**Keywords:** Orthorexia nervosa, Diagnosis, Clinical utility, Eating disorders

## Abstract

**Purpose:**

This study examined U.S. health professionals’ perspectives on the clinical utility, measurement, and etiology of orthorexia nervosa (ON).

**Methods:**

Participants (*n* = 100) were U.S. health professionals with experience working clinically with eating disorders, including trainees, Ph.D. psychologists, social workers/mental health counselors, and medical health professionals. After reviewing the proposed ON criteria, participants responded to questions regarding the clinical utility, diagnosis, and measurement of ON, and sociocultural influence on the emergence of ON. Views of ON as a useful diagnostic category were examined as a function of participants’ current involvement in clinical versus research activities.

**Results:**

Participants mostly (71.9%) agreed that ON should be a distinct clinical diagnosis. Participants who endorsed ON as a valid diagnosis spent more time on clinical work and less time engaged in research compared to participants who disagreed (both ps < 0.05). Approximately 27% of participants believed additional components should be added to the proposed ON diagnostic criteria. Participants indicated that sociocultural factors have considerable influence on the development of ON, namely the diet and weight loss industry, and the perceptions that biological/organic/vegan and low fat/low carb/gluten free food are the healthiest.

**Conclusion:**

Professionals who spent more time working clinically with eating disorders were more likely to endorse ON as a unique disorder, and professionals who spent more time on research were more likely to disagree. To the extent that professionals who spend more time on research may shape the narrative around ON more visibly, this study underscores the importance of listening to practitioners' experiences in applied settings.

*Level of evidence:* Level V: Opinions of authorities, based on descriptive studies, narrative reviews, clinical experience, or reports of expert committees.

**Supplementary Information:**

The online version contains supplementary material available at 10.1007/s40519-023-01551-6.

## Introduction

Orthorexia nervosa (ON), a condition that is characterized by a pathological obsession with eating healthy food [1], has been widely described in the scientific literature and popular press, but has not been formally recognized as a disorder in either the Diagnostic and Statistical Manual of Mental Disorders (DSM) [2] or the International Classification of Diseases (ICD) [3]. An ongoing debate as to whether ON is a unique eating disorder (ED) inhibits rigorous research on the condition. An important contribution to this debate is the opinions of practicing health professionals with experience working clinically with ED patients. While recent studies have examined professionals’ opinions of ON as a meaningful diagnosis outside of the U.S. [4–6], knowledge of U.S. health professionals’ opinions on this matter is lacking. Given that the DSM and ICD are the standard classifications of mental disorders used by mental health professionals and insurance companies in the U.S., specifically surveying U.S. health professionals is a critical extension of prior work. The lack of consensus on the clinical utility, diagnostic criteria and measurement, and sociocultural influence of this disordered eating pattern cause problems for the interpretation of much of the ON literature.

## Clinical utility

Clinical utility refers to a diagnostic concept whose defining features provide useful information such as prognosis, likely treatment outcomes, and/or testable propositions about biological correlates [7]. Prior surveys of clinicians working with ED patients in Belgium, the Netherlands, Australia, and New Zealand found that a majority reported they had observed ON in their own practice and thought it should be a distinct, clinically recognized disorder [4–6].

A key consideration regarding the clinical utility of ON is whether it constitutes a meaningful presentation that is distinct from other EDs. Generally, the main arguments against ON involve consideration of the disordered eating pattern as being better accounted for by existing diagnoses. Similar to other EDs, perfectionism, obsessive–compulsive traits, general psychopathology, dieting, poor body image, and drive for thinness have been shown to be positively associated with greater ON symptoms [8]. In contrast, although weight and shape concerns are considered to be integral to the psychopathology of AN and BN [9], their relevance is less clear for ON. In the original criteria proposed by Dunn and Bratman [10], dietary restriction to promote optimum health may lead to weight loss for individuals with ON, but the desire to lose weight is *absent, hidden or subordinated to ideation about healthy eating*. Extant research supports this proposed criterion, finding a strong preoccupation with healthy eating to be negatively correlated with overweight preoccupation and appearance evaluation in a sample of university students [11]. However, a recent study compared ON symptoms to ED and symptoms and found that not only are ON symptoms related to body weight and shape concerns, but also ON symptoms correlated with prioritizing weight above health with respect to food selection [12]. ON has also been proposed to fall under the Obsessive Compulsive and Related Disorders category of the DSM-5, mainly due to symptom overlap with obsessive–compulsive disorder [9, 13]. Indeed, in a sample of U.S. college students, ON was significantly associated with obsessive–compulsive behaviors [14].

## Diagnosis and measurement

Currently, four sets of diagnostic criteria for ON have been proposed [10, 15–17] and four ON assessments have typically been used in past research [1, 16, 18, 19]. The lack of consensus for defining and measuring the proposed construct results in significant issues with research on ON. Further, the assessments of ON that are currently available and used in research have been criticized for low internal consistency, questionable reliability, and incomplete assessment of proposed criteria [20]. Yet, one of these measures, the ORTO-15, continues to be used in research, resulting in unreliable and problematic findings. Prevalence ratings for ON as measured by the ORTO-15 have been found to be as high as 81.9% [21], evidently over-pathologizing what are most likely normative food choice behaviors. Furthermore, the ORTO-15 produces a wide range of prevalence rates. For example, using the ORTO-15 criteria, one study found a prevalence rate of ON of 71% among U.S. college students; however, when they considered whether participants’ diets had led to impairment in everyday living or medical problems, they reported ON rates as less than 1% [22].

Current diagnostic criteria and assessment tools for ON have been criticized for not comprehensively measuring what some believe to be distinct components of the proposed disordered eating pattern. For example, compulsive or excessive exercise has been linked to ON symptomatology in previous studies [23–25], suggesting that the addition of this behavior to the diagnostic criteria or assessment for ON may capture the phenomenon more comprehensively. However, it is important to note that excessive exercise is not considered a diagnostic criterion in anorexia nervosa (AN) despite significant co-occurrence [2, 26]. In addition, a clearer criterion regarding the role of weight or shape concerns in ON would be helpful, given the inconsistency of existing literature on the topic [11, 12]. Other additional criteria specifically intended to differentiate ON from other related disorders would be beneficial for the measurement of ON.

## Sociocultural factors contributing to the emergence of ON

In addition to considerations regarding clinical utility and measurement and diagnostic issues, there is debate about etiologic trajectories that may potentially be unique or specifically relevant to our understanding of ON. Particular attention in the literature has been directed toward the influence of sociocultural factors on the emergence of ON [27, 29]. The rising popularity of health and fitness and the ubiquity of social media are two areas of focus within the sociocultural lens. The modern fixation on “healthy eating” can be traced back to the late-nineteenth early-twentieth century popularization of different diet and weight loss practices, spearheaded by companies such as Weight Watchers and Atkins that are still recognizable names today. Obesity became a salient concern in the mid-twentieth century, and it brought a transformation in the rationale for weight loss from individual goals to societal responsibility to combat rising rates [30]. This societal pressure to be thin resulted in the conceptualization of “healthism”, or the idea that the responsibility to prevent disease relies on the individual and their choice to change their own circumstances [31]. “Healthism” made a person’s weight synonymous with their health and well-being, and solidified the moralization of food choice or process by which preferences are converted into a marker of values and perceived individual morality, that perpetuates modern ideas today [31, 32].

The growing popularity of social media platforms has also been hypothesized as contributing to the emergence of ON [33]. In a content analysis of the social media platform Instagram posts designed to promote fitness, results suggest that the majority of pictures portrayed thin and toned females, often with objectifying elements [34]. Although ostensibly promoting health and well-being, the characteristics associated with these posts, such as the thin body ideal, over-emphasis on appearance, and objectification have been linked to poorer body image and disordered eating. Indeed, one study found that higher Instagram use was associated with higher scores on the ORTO-15, indicating a greater tendency towards ON-related symptoms [28].

Results from prior studies outside of the U.S. suggest that many health professionals attribute sociocultural factors to the emergence of ON [29], although less is known about how health professionals in the U.S. conceptualize this disordered eating condition. Understanding how sociocultural factors may influence the development of ON, and which factors (e.g., social media) are most impactful, can inform prevention and treatment for ON that may differ slightly from the conceptualization of sociocultural factors related to risk in other EDs.

## Current study

We aimed to replicate and expand on prior studies [4, 29] by assessing U.S. health professionals’ perspectives on: (1) ON as a distinct, clinically recognized disorder, (2) best practices in measurement and diagnosis of ON, and (3) supports for the idea that various sociocultural factors may be linked to the emergence of ON. Findings will represent an important contribution to the ongoing debate about possible inclusion of the proposed ON diagnosis in the next iteration of the DSM, and for our understanding of this condition.

## Methods

All study procedures were approved by the appropriate Institutional Review Boards. Participants reviewed a consent form describing the nature and purpose of the research prior to completion of questionnaires.

### Procedures

All participants were recruited through social media advertisements (Facebook, Instagram, and Twitter, as well as professional listservs (e.g., the Academy for Eating Disorders)) that included a link to the survey (Supplementary Information) hosted on the secure server Qualtrics. Inclusion criteria were being a health professional with experience working with EDs in the United States.

### Participants

Participants were health professionals (*n* = 100) with experience working clinically with EDs. Four participants were excluded from analyses because they had not worked clinically with EDs before, resulting in a final sample size of 96 (mean age = 35.54 years, SD = 9.78, range: 21–68 years; 92.70%^1^ female; 93.80% White). The sample included PhD level psychologists (32.30%), graduate and post-baccalaureate trainees (25.00%), social workers/mental health counselors (24.00%), and medical health professionals (Psychiatrists and other Physicians., Registered Dieticians, Registered Nurses, Physician Assistants,, and Nurse Practitioners; 18.80%). Participants reported an average of 8.83 years practicing in their professions (*SD* = 8.19, range 1–36 years).

### Measures

The questionnaire administered in the present study was adapted from Ryman et al. [4] and Syurina et al. [29] to facilitate direct comparisons between the current and prior samples. Minor modifications or additions were made as detailed below.

#### Clinical utility of ON

To begin the survey, participants were presented with the proposed diagnostic criteria for ON [10] and asked if they had met clients who fulfilled these criteria. We added, “What diagnosis/diagnoses did you give to clients who fulfilled these criteria?” to the original survey questions, in order to clarify how clinicians are currently categorizing this disordered eating pattern in their practice. Participants were then asked how prevalent they believed the condition was in the general population in the U.S., and if they believed ON should have its own diagnosis in the upcoming version of the DSM (via “yes/no” responses).

#### Diagnosis and measurement of ON

Participants were asked which diagnostic category/ies they believed ON would fall under in the DSM and were asked to rate, on a scale from 1 (*not at all a contributor*) to 5 (*a vital contributor*), the extent to which exercise and weight loss contribute to or are a part of ON. They also responded to the following yes/no questions: “Do you think exercise related symptoms should be part of the diagnostic criteria?” and “Are there any additional components that are missing from the diagnostic criteria?” followed by an open-ended question to describe the components they proposed. These questions were added to the original study based on recent literature critiquing the proposed criteria [20], and proposing additional relevant criteria for the ON diagnosis [16].

#### Sociocultural factors contributing to the emergence of ON

As was done in the original studies, participants were asked to what extent they believed certain worldviews and values, markets and industries, mass media and beauty ideals influenced the emergence of ON, which each potential factor being rated on a scale from 1 (*no influence at all*) to 5 (*great influence*). For the purposes of the current study, we changed the language of each question from “to what extend do you consider each of the following *parts of the modern Western culture* to influence the emergence of Orthorexia” to “to what extent do you consider each of the following *sociocultural factors*^2^ to influence the emergence of Orthorexia.” We also expanded the original survey’s questions about the influence of digital media by asking about social media separately from other digital media.

#### Demographics

The last section of the survey included questions on gender, age, race/ethnicity, country of residence, highest level of education, profession, experience working clinically with EDs (indicated via yes/no response), and the percentage of professional time spent on research and clinical work.

### Statistical analyses

Descriptive analyses and independent samples *t*-tests were conducted to determine average prevalence estimates for ON and to examine differences in the opinions of ON as a discrete diagnosis (yes/no) by participants’ professional time (as a percentage of total) allocated to research versus clinical work. Participants’ descriptions of additional components they believed should be incorporated into the ON criteria were categorized by two independent raters with ED knowledge (90.48% initial inter-rater agreement; a third rater resolved the remaining two discrepancies). Total scores on the sociocultural factors related to ON scale were calculated for each participant, with higher scores indicating more agreement that sociocultural factors have a major influence on ON (Cronbach’s *α* = 0.88). Because we added an additional item assessing perceived influence of social media, total score percentages were calculated to allow for comparisons across samples. Bivariate correlations were used to examine any differences in perceived sociocultural influence by participants’ professional time allocated to research versus clinical work. Due to the nature of the community sample and lack of compensation for participation, missing data are found throughout, and valid percent is always reported.

## Results

### Clinical utility of ON

Most participants (78.1%) endorsed previously working with individuals whom they considered to fulfill the proposed ON criteria. Participants indicated that they typically diagnosed these clients with AN (47.9%), Other Specified Feeding or Eating Disorder (OSFED)/Eating Disorder Not Otherwise Specified (EDNOS) (28.1%), and Generalized Anxiety Disorder (22.9%). Participants indicated moderate perceived prevalence of ON in the U.S. general population (*M* = 3.05, SD = 1.02 on a scale from 1 = *not at all prevalent* to 5 = *extremely prevalent*). Most participants agreed ON should be included as a diagnosis in future versions of the DSM (71.9%). Of respondents who reported that ON should not be considered a separate diagnosis (28.1%), the majority believed the disordered eating pattern fit within AN (18.8%), Obsessive Compulsive Disorder (OCD) (10.4%), OSFED/EDNOS (7.1%), or Avoidant Restrictive Food Intake Disorder (7.1%). Compared to those who reported ON should be recognized as a unique disorder, the health professionals that disagreed reported spending significantly more time on research and significantly less time on clinical work (Table 1). No significant differences in views of ON emerged when examining results by participants’ professions.

**Table 1:** Percentage of time spent on research and clinical work for participants who did and did not endorse ON as a discrete diagnosis

	ON-No		ON-Yes		*df*	*t*	*p*	Cohen’s *d*
	*M*	SD	*M*	SD				
Clinical work	41.69	28.51	63.31	34.37	82	2.85	< 0.05	0.68
Research	54.12	29.68	36.69	30.59	82	2.44	< 0.05	0.59

ON-No describes the participants who disagreed that ON should have a formal diagnosis in the upcoming DSM. ON-Yes describes the participants that believed ON should have a formal diagnosis in the upcoming DSM. *M* represent the mean percentage (out of 100%)

### Diagnosis and measurement of ON

Almost all respondents (94.8%) reported that ON fit within the DSM category of “Eating and Feeding Disorders;” fewer believed it should fall under “Obsessive Compulsive” (36.5%) or “Anxiety Disorders” (14.6%). Weight loss (*M* = 3.42, SD = 1.14, on a scale from 1 = *no influence at all* to 5 = *great influence*) and exercise (*M* = 3.49, SD = 1.17) were thought to be moderately influential contributing factors to ON; 62.1% of participants agreed that exercise-related symptoms should be part of the ON diagnostic criteria. Twenty-five participants (26.6%) believed additional components should be added to Dunn and Bratman’s [10] proposed ON diagnostic criteria. Besides the exercise-related symptoms, participants (*n* = 4) identified body shape/weight concerns as an important additional component that needs to be highlighted in the criteria. For example, one participant noted:

*“Weight is often a concern (such as avoiding weight gain) even if not the primary concern. Folks are rarely neutral about weight.”*

Relation to disease prevention or general health concerns was noted as worth emphasizing in diagnostic criteria (*n* = 5):

*“Perhaps mention that the restriction in eating may have originally stemmed from a medical concern/health condition (e.g., diabetes), but over time has progressed such that the food choices become much more restrictive than is medically necessary for the condition.”*

Additional open-ended responses included information on how to differentiate ON from other disorders such as AN and OCD, as well as rejection of scientific or medical information that contradicts patients’ beliefs and fears of eating certain foods (Supplementary Information).

### Sociocultural factors contributing to the emergence of ON

The total mean score percentage on the sociocultural influence ratings in this sample was 78.8% (SD = 0.10, range: 40–96%). Participants rated the diet and weight loss industry, and the perceptions that biological/organic/vegan and low fat/low carb/gluten free food are the healthiest as the most influential sociocultural factors in the emergence of ON (see Table 2 for descriptives).

**Table 2:** Mean scores and SD for each item in the sociocultural factors questionnaire

Sociocultural factor	Mean	SD
Individualism	3.10	1.24
Materialism	3.10	1.30
Capitalism	3.52	1.34
Food industry	4.23	0.92
Diet and weight loss industry	4.70	0.63
Fitness industry	4.64	0.64
Fashion industry	3.76	1.16
Cosmetic surgery industry	3.51	1.22
Broadcast media	4.01	0.86
Social media	4.63	0.60
Other digital media	3.94	0.87
Printed media	3.45	1.00
Outdoor advertisements	3.02	0.92
Thin body ideal	4.25	0.93
Muscular body ideal	4.16	0.95
Fast food is unhealthy	4.51	0.81
Biological/organic/vegan food is the healthiest	4.66	0.64
Low fat/low cab/gluten free food is the healthiest	4.66	0.57
Regular exercise is best for the body	3.76	1.08
Eating fast food	2.99	1.25
Trends of having healthy diets	4.47	0.85

Perceived sociocultural influence on ON was significantly and positively correlated with time allocated to clinical work (*r* = 0.25 *p* < 0.05), but unrelated to time allocated to research (*r* = − 0.20, *p* = 0.06). No significant differences in perceived sociocultural influence on ON emerged for the different professions. In response to the question, “do you think there are any types of digital media in particular that influence the emergence of Orthorexia?” Instagram was specifically mentioned by 64 participants, whereas 26 participants identified Tik Tok, 12 mentioned Facebook and seven mentioned Twitter as particularly influential on the emergence of ON. For example, one participant responded:

*“Social media in general (Facebook, TikTok, Instagram, Twitter) have all been labeled by patients I have treated in the past year as sources of inspiration and guidance on eating in orthorexic patterns.”*

## Discussion

This study was the first, to our knowledge, to assess U.S. health professionals’ perspectives on ON as a disordered eating pattern. Recent studies reported health professionals’ opinions of ON outside of the U.S, specifically in the Netherlands [4], Australia and New Zealand [5], and Belgium [6]. Findings from the current study give insight into the current state of knowledge and beliefs among practicing health professionals in the ongoing debate about ON.

Similar to studies previously conducted in the Netherlands, Australia and New Zealand, a majority of the U.S. health professionals surveyed here (72%) indicated that ON should be included as a diagnosis in future versions of the DSM. Contrary to the original study [4], we did not find differences in perspectives about ON by profession, potentially due to small subsample sizes (e.g., ns = 31 or less in each category). However, differences did emerge when we examined types of work within professions, namely professional time allocated to research and clinical work. Health professionals who spend more time conducting clinical work were more likely to endorse ON as a discrete diagnosis compared to participants who reported spending more time on research. The disparate opinions of those who engage in more clinical work compared to research raises an important consideration of how the ON diagnosis debate translates from academia to clinical work and by association, to the individuals suffering from this disordered eating pattern. Specifically, the current study highlights a contrast between professionals who see many ED patients but whose opinions and experiences may not be as visible and professionals who shape the narrative around ON more saliently through research and publications.

Consistent with prior work [4], a majority of participants in the current study reported that ON would fit under the DSM category Eating and Feeding Disorders and many agreed that exercise-related symptoms should be part of the diagnostic criteria for ON. Qualitative data provided several interesting considerations for additional components to the proposed ON criteria. Participants in the current study noted that weight in some sense is often a concern for the ON patients they've encountered, and suggested body shape/weight concerns should be considered more explicitly in the criteria. Some health professionals in the current study also believed that the fear of eating certain foods warranted placement in the criteria. Lastly, participants emphasized the importance of recognizing the disease prevention and general health concerns often present for individuals with ON symptoms. With the exception of exercise-related symptoms, perspectives of the health professionals in the current study largely align with those of a multidisciplinary expert panel that recently published a consensus document on the definition and diagnostic criteria for ON [35].

Finally, the current study provides both quantitative and qualitative data to support the significant influence of sociocultural factors in the emergence of ON, according to the opinions of health professionals with experience working with EDs. Our findings are near direct replications of the original study [29] emphasizing the two perceptions that biological/organic/vegan food and low fat/low carb/gluten free food are the healthiest as the most influential factors in the emergence of ON. Our findings add to prior work by highlighting the significant perceived role of social media in the etiology of ON, an area of study that merits further attention in future work. More professional time spent on clinical work was associated with greater perceived sociocultural influence on the emergence of ON, providing further rationale for researchers to listen to practitioners’ experiences in applied settings in order to work toward a consensus regarding risk and maintaining factors for ON.

## Limitations and future directions

This is the first study, to our knowledge, to qualitatively investigate health professionals' perspectives on the specific components of the diagnostic criteria for ON proposed by Dunn and Bratman [10]. Although the information provided by participants contribute to the ongoing discussion about ON, some important limitations must be noted. Participants were recruited via social media channels and may represent a subgroup of health professionals who use social media regularly, thus limiting generalizability of findings. Though we were able to recruit a diverse sample in terms of profession, age, and years of experience, generalizability of findings remains limited due to the fact that the sample was majority White and female.

Our findings support recent efforts to conceptualize ON within a broader category of EDs dimensionally, rather than a distinct category. Indeed, much of the ON debate reflects the continued struggle with discrete diagnostic categories of overlapping eating pathology that are still necessary for research and insurance purposes, yet less meaningful in applied clinical work. In some ways, the validity of ON as a unique disorder relative to other EDs may be less relevant than its usefulness to clinicians attempting to conceptualize an individual’s current presentation as accurately as possible. Kendell and Jablensky [7] conceptualize usefulness of psychiatric disorders in two parts: first, the quantity and quality of the information in the literature, which depend on adequate diagnostic criteria not currently present for ON; second, whether the implications of the etiological, prognosis, and treatment information are different from the implications of other related syndromes. In the case of ON, perhaps sociocultural factors are more primary considerations for preventing and treating this presentation compared to other EDs (e.g., with social media literacy training). Further, Kendell and Jablensky [7] describe psychiatric diagnoses as working concepts that are invaluable to clinicians because of their utility rather than their validity. The aforementioned review of the ON literature and data provided by the current study support the notion that ON is a working concept with explicit support from health professionals internationally.

## Conclusions

Overwhelmingly, U.S. health professionals with experience working with EDs endorsed the opinion that ON should have its own diagnosis in the upcoming version of the DSM, and many participants provided qualitative responses suggesting this opinion was based on their clinical experience with individuals suffering with ON-like symptoms. Although no differences emerged by profession as in prior studies, the type of work performed as a health professional was associated with whether participants agreed or disagreed that ON should be a distinct disorder. The ultimate goal of any health professional working with ED patients is to alleviate the suffering caused by the disorder. Therefore, understanding why differences in opinions emerged based on professional time allocated to research and clinical work is important in order to best discern how to work together as health professionals in the ED field toward this mutual goal. Given the majority opinion that an ON diagnosis would be useful for health professionals in the current sample and prior studies, continued work toward reliable measurement and assessment of the condition is warranted.

## Supplementary Information

Below is the link to the electronic supplementary material.

Supplementary file1 (PDF 173 KB)

Supplementary file2 (PDF 108 KB)

## Footnotes

1 Given that our N is approximately 100, only percentages are presented throughout.

2 We chose to modify the original survey’s language because “sociocultural factors” is much broader than “modern Western culture” and may include factors and variables that perhaps are not specific or unique to what is traditionally referred to as "Western culture”.
