# Supplementary material for: U.S. health professionals’ perspectives on orthorexia nervosa: clinical utility, measurement and diagnosis, and perceived influence of sociocultural factors
Source: Eat Weight Disord. 2023 Mar 22;28(1):31. doi: 10.1007/s40519-023-01551-6 (PMC10033613; doi:10.1007/s40519-023-01551-6)
Supplement: Supplementary file 1 — Supplementary file1 (PDF 173 KB) [file 40519_2023_1551_MOESM1_ESM.pdf]

Supplementary Information

U.S. Health Professionals' Perspectives on Orthorexia Nervosa: Clinical Utility, Measurement and Diagnosis, and Perceived Influence of Sociocultural Factors

Eating and Weight Disorders

Christina M. Sanzari, MA & Julia M. Hormes, Ph.D.
University at Albany, State University of New York, csanzari@albany.edu

Questionnaire

Orthorexia Nervosa is an emerging disordered eating pattern, in which the individual acquires a pathological obsession with healthy eating. This can have physical, psychological and social consequences.

The aim of this survey is to shed light on the perspectives of health professionals on the condition, current diagnostic criteria, possible influential factors and how Orthorexia Nervosa should be classified.

The survey will consist of three chapters:
Chapter 1: Recognition, diagnosis, and classification of Orthorexia.
Chapter 2: Influence of modern Western culture on Orthorexia.
Chapter 3: Characteristics of respondent.
Overall, it will take approximately 10 min to fill in the questionnaire. You may have additional thoughts about these questions, and you will have space at the end to write in any additional comments.

Chapter 1

Please read the proposed diagnostic criteria for Orthorexia Nervosa carefully in order to accurately answer the following questions.

**Criterion A**	Obsessive focus on “healthy” eating, as defined by a dietary theory or set of beliefs whose specific details may vary; marked by exaggerated emotional distress in relationship to food choices perceived as unhealthy; weight loss may ensue as a result of dietary choices, but this is not the primary goal. *As evidenced by the following:*
**A1.**	Compulsive behavior and/or mental preoccupation regarding affirmative and restrictive dietary practices believed by the individual to promote optimum health
**A2.**	Violation of self-imposed dietary rules causes exaggerated fear of disease, sense of personal impurity and/or negative physical sensations, accompanied by anxiety and shame.
**A3.**	Dietary restrictions escalate over time, and may come to include elimination of entire food groups and involve progressively more frequent and/or severe “cleanses” (partial fasts) regarded as purifying or detoxifying. This escalation commonly leads to weight loss, but the desire to lose weight is absent, hidden or subordinated to ideation about healthy eating.
**Criterion B**	The compulsive behavior and mental preoccupation becomes clinically impairing by *any of the following*:
**B1.**	Malnutrition, severe weight loss or other medical complications from restricted diet
**B2.**	Intrapersonal distress or impairment of social, academic or vocational functioning secondary to beliefs or behaviors about healthy diet
**B3.**	Positive body image, self-worth, identity and/or satisfaction excessively dependent on compliance with self-defined “healthy” eating behavior

Have you met clients who fulfill these criteria?

○ Yes, within the last year

○ Yes, more than one year ago

○ No

What diagnosis/diagnoses did you give to clients who fulfilled these criteria? You can select more than one option.

☐ Anorexia Nervosa

☐ Bulimia Nervosa

☐ Avoidant/Restrictive Food Intake Disorder

☐ Obsessive Compulsive Disorder

☐ Generalized Anxiety Disorder

☐ Other (please specify)

____

How prevalent do you think the condition is in the general population in the United States, with 1 meaning not at all prevalent and 5 meaning extremely prevalent?

○ 1 (not at all prevalent)

○ 2

○ 3

○ 4

○ 5 (extremely prevalent)

Do you think Orthorexia Nervosa should have its own diagnosis in the upcoming versions of the DSM?

○ Yes

○ No

Which existing diagnosis do you think it fits within? You can select more than one option.

- ☐ Anorexia Nervosa
- ☐ Bulimia Nervosa
- ☐ Avoidant/Restrictive Food Intake Disorder
- ☐ Obsessive Compulsive Disorder
- ☐ Generalized Anxiety Disorder
- ☐ Other (please specify)

____

In the DSM-5, diagnoses are divided into different categories of diseases or disorders of the same type.
Which diagnostic category or categories does Orthorexia Nervosa fall under according to your opinion? You can select more than one option.

- ☐ Eating and Feeding Disorders
- ☐ Obsessive Compulsive Disorders
- ☐ Anxiety Disorders
- ☐ Other (please specify):

____

In the media, several factors are presented as possible contributors or part of the condition.

On a scale from 1 to 5, to what extent do you think each of the following factors contribute to/are part of Orthorexia Nervosa (1 meaning not a contributor/not part of the condition at all and 5 meaning a vital contributor/great part of the condition)

	1 (not a contributor)	2	3	4	5 (a vital contributor)
Exercise	○	○	○	○	○
Weight loss	○	○	○	○	○

Do you think exercise related symptoms should be part of the diagnostic criteria?

○ Yes

○ No

Are there any additional components that are missing from the diagnostic criteria?

○ Yes (please explain) ____

○ No

Chapter 2 Influence of sociocultural factors on Orthorexia

In this chapter you will get questions regarding a possible influence of sociocultural factors on the emergence of Orthorexia. This questionnaire will not ask about other factors that could influence orthorexia, but the fact that they are prevalent is acknowledged

On a scale of 1–5, with 1 meaning no influence at all and 5 meaning a great influence, to what extent do you consider each of the following sociocultural factors to influence the emergence of Orthorexia?

	1 (no influence at all)	2	3	4	5 (a great influence)
Individualism	○	○	○	○	○
Materialism	○	○	○	○	○
Capitalism	○	○	○	○	○
Food industry	○	○	○	○	○
Diet- and weight loss industry	○	○	○	○	○
Fitness industry	○	○	○	○	○
Fashion industry	○	○	○	○	○
Cosmetic surgery industry	○	○	○	○	○
Broadcast media (Television, movies, and radio)	○	○	○	○	○
Social Media	○	○	○	○	○
Other Digital Media (Internet websites)	○	○	○	○	○
Printed media (Books, newspapers, and magazines)	○	○	○	○	○

Outdoor advertisements (Billboards, shops, etc.)	○	○	○	○	○

On a scale of 1–5, with 1 meaning no influence at all and 5 meaning a great influence, to what extent do you consider the following sociocultural <u>perceptions and behaviors</u> to influence the emergence of Orthorexia?

	1 (no influence at all)	2	3	4	5 (a great influence)
Thin body ideal	○	○	○	○	○
Muscular body ideal	○	○	○	○	○
Fast food is unhealthy	○	○	○	○	○
Biological/organic/vegan food is the healthiest	○	○	○	○	○
Low fat/low carb/gluten free food is the healthiest	○	○	○	○	○
Regular exercise is best for the body	○	○	○	○	○
Eating fast food	○	○	○	○	○
Trends of having healthy diets	○	○	○	○	○

Do you think there are any types of digital media in particular that influence the emergence of Orthorexia (e.g., Facebook, Instagram, Twitter)? Please explain.

____

Chapter 3: Characteristics of Respondent
This chapter consists of questions regarding your characteristics.

What gender do you currently identify with?

○ Male

○ Female

○ Other ____

○ Prefer not to say

What is your age in years?

____

What is your ethnicity/race? You can select more than one option.

☐ White

☐ Black or African American

☐ Asian

☐ Native Hawaiian or Pacific Islander

☐ Native American or Alaskan Native

☐ Hispanic/Latino

☐ Other ____

Where do you live currently?

○ United States

○ Outside of the United States

What is your highest completed level of education?

◯ Bachelor's Degree

◯ Master's Degree

◯ PhD/PsyD

◯ Other ____

What is your profession?

____

For how many years have you been practicing this profession?

____

Have you encountered patient(s) with a suspected or confirmed eating disorder in the profession?

◯ Yes

◯ No

If applicable, what percent of time do you spend on the following areas in your profession?
Note: depending on other obligations, these do not have to add up to 100.

	0 10 20 30 40 50 60 70 80 90 100
Research	
Clinical work	
