# Supplementary material for: U.S. health professionals’ perspectives on orthorexia nervosa: clinical utility, measurement and diagnosis, and perceived influence of sociocultural factors
Source: Eat Weight Disord. 2023 Mar 22;28(1):31. doi: 10.1007/s40519-023-01551-6 (PMC10033613; doi:10.1007/s40519-023-01551-6)
Supplement: Supplementary file 2 — Supplementary file2 (PDF 108 KB) [file 40519_2023_1551_MOESM2_ESM.pdf]

Supplementary Information

U.S. Health Professionals' Perspectives on Orthorexia Nervosa: Clinical Utility, Measurement and Diagnosis, and Perceived Influence of Sociocultural Factors
Eating and Weight Disorders
Christina M. Sanzari, MA & Julia M. Hormes, Ph.D.
University at Albany, State University of New York, csanzari@albany.edu

Participants Responses to The Question, "Are There Any Additional Components That Are Missing from The Diagnostic Criteria?"

Theme	Participant Response
**body weight/ shape concerns**	Focus on muscularity for men and leanness for women
	weight is often a concern (such as avoiding weight gain) even if not the primary concern. Folks are rarely neutral about weight.
	Usually hard to weight restore
	The absence of weight loss/weight concerns is confusing to me as I would assume part of the motivation for 'healthy eating' would be weight control or maintaining an 'ideal' body shape/weight.
**Fear of eating certain foods**	Selective eating/fear of eating certain things
	Fears related to specific types of foods
	Despite being informed by professionals that their fear/beliefs about foods is unfounded, continue to maintain behaviors that endorse ON
**Disease prevention/ general health concerns**	Preoccupation with health related concerns in general - several patients i've seen describe that their behaviors are in effort to avoid cancer diagnoses.
	A description of overall obsession related to body "toxins" - eg medications, vaccines, pesticides, bpa
	Perhaps mention that the restriction in eating may have originally stemmed from a medical concern/health condition (e.g., diabetes), but over time has progressed such that the food choices become much more restrictive than is medically necessary for the condition.
	Consider disease prevention and health anxiety as one of the desired outcomes of eating healthy

	regarding exercise, as I understand ON, I do not think this needs to be included as a required criterion, but could be included as an example of a hyperactive "healthy" behavior
**Other responses**	Factors that differentiate ON from OCD and AN
	Guilt as well as shame
	trying to convince others to follow their rules (as opposed to the secretiveness seen with AN or BN); rejection of scientific or medical information that contradicts their beliefs
	Refusal to eat when food is not available that abides by their values
	Something regarding drastic changes in diet based on new information (fad diets)
	Clarifying the function of the behavior(s) to better distinguish it from other disorders. Is it to reduce anxiety? Is it to control weight or shape? Is it based of health beliefs?
	cultural considerations
	objective medical data
	Not missing per se, but just highlighting that weight loss is not the goal - this reminds me of ARFID. Could be listed as a feeding disorder, rather than an ED...
